# Water use strategies of *Ferula bungeana* on mega-dunes in the Badain Jaran Desert

**DOI:** 10.3389/fpls.2022.957421

**Published:** 2022-12-06

**Authors:** Jie Qin, Jianhua Si, Bing Jia, Chunyan Zhao, Dongmeng Zhou, Xiaohui He, Chunlin Wang, Xinglin Zhu

**Affiliations:** ^1^ Key Laboratory of Eco-Hydrology of Inland River Basin, Northwest Institute of Eco-Environment and Resources, Chinese Academy of Sciences, Lanzhou, China; ^2^ University of Chinese Academy of Sciences, Beijing, China

**Keywords:** stable isotope, water uptake, plant water potential, plant hydraulic conductivity, water use efficiency

## Abstract

In desert ecosystems, ephemeral plants have developed specialized water use strategies in response to long-term natural water stress. To examine the water use strategies of desert ephemeral plants under natural extreme drought conditions, we investigated the water absorption sources, water potential, hydraulic conductivity, and water use efficiency of *Ferula bungeana* at different elevations on the slopes of mega-dunes in the Badain Jaran Desert, Inner Mongolia, during a period of extreme drought. We found that the water utilized by *F. bungeana* was mostly absorbed from the 0–60 cm soil layers (80.47 ± 4.28%). With progression of the growing season, the source of water changed from the 0–30 cm soil layer to the 30-60 cm layer. The water potentials of the leaves, stems, and roots of *F. bungeana* were found to be characterized by clear diurnal and monthly variation, which were restricted by water availability and the hydraulic conductivity of different parts of the plant. The root hydraulic conductivity of *F. bungeana* was found to be considerably greater than that of the canopy, both of which showed significant diurnal and monthly variation. The water use efficiency of *F. bungeana* under extreme drought conditions was relatively high, particularly during the early and late stages of the growing season. Variations in water availability led to the regulation of water uptake and an adjustment of internal water conduction, which modified plant water use efficiency. These observations tend to indicate that the water use strategies of *F. bungeana* are mainly associated with the growth stage of plants, whereas the distribution pattern of plants on mega-dunes appeared to have comparatively little influence. Our findings on the water use of ephemeral plants highlight the adaptive mechanisms of these plants in desert habitats and provide a theoretical basis for selecting plants suitable for the restoration and reconstruction of desert ecosystems.

## Introduction

Desert ecosystems cover approximately 20% of global land area, and 43% of land is under threat of desertification. In recent years, global warming and increased human disturbances (IPCC, 2019) have markedly affected the accessibility of water resources in desert ecosystems. The sandy surface of the desert oasis transition zone has been activated, the vegetation protection system degraded, and the ecological environment has undegone a notable deterioration. In particular, during the 20^th^ century, the Sahara Desert is estimated to have expanded by more than 10% (Thomas and Nigam, 2018). Similarly, the extent of the Badain Jaran Desert in Inner Mongolia increased by 363.24 km^2^ in the 30 years between 1990 and 2018 (Wang, 2019). Given such trends, the restoration of desert ecosystems has emerged as an urgent problem in recent decades, and our efforts to control desertification have a long way to go. The most fundamental and effective measure for stemming the increase in desertification entails the control and fixation of drifting sand by establishing vegetation, and in this regard, it is of particular importance to select the most appropriate plant species for the ecological restoration or reconstruction of desert ecosystems. Unless such measures are taken, the extensive expansion of deserts and intensification of desertification will have severe repercussions for continued human subsistence.

When screening plant species for the suitability for use in ecosystem restoration or reconstruction, a primary consideration is their ability toadapt to water stress. In this regard, the water use strategy of plants can fully reflect the adaptability of plants to changing moisture environments (Dawson et al., 2002; Evaristo et al., 2016; Grossiord et al., 2017), and determine the mechanisms and extents of plant responses to hydrological change (Antunes et al., 2018a; Wu et al., 2019). Plants growing in arid regions adapt to water scarcity by modifying their physiological characteristics and reproductive stratgies, and respond to different water sources by adopting corresponding water use strategies. Whereas annuals are completely dependent on water obtained from summer precipitation, perennials can survive periods of long-term drought predominantly by the ability of their root systems to obtain the remaining soil moisture and the capacity of the above-ground organs to withstand water stress (Ehleringer et al., 1991). The efficiency with which plant roots obtain water is strongly dependent on the soil water availability, rooting depth, root distribution, and root hydraulic properties (Zencich et al., 2002; Warren et al., 2015; Phillips et al., 2016). Among these factors, the availabilityof soil water is a key determinant of plant growth and survival (Antunes et al., 2018b). During periods of drought, rooting depth primarily determines how much water plants can extract from the soil (Zencich et al., 2002), whereas during periods when the soil is occupied with water, the breadth of roots close to the soil surface is the principal determinant of water uptake. Root activity determines the process of root water uptake (Wu et al., 2014; Warren et al., 2015; Phillips et al., 2016; O'Keefe et al., 2019), and during “wet” periods, roots in the lateral root zone take up surface water, whereas when shallow water sources are insufficient, plants are dependent on the activity of penetrating taproots that gain access to deep soil water. Accordingly, differences in root morphology lead to different patterns of water uptake in plants (Wang et al., 2019; Wu et al., 2019), with shallow-rooted plants generally exploiting shallow soil water sources, and species with dimorphic root systems typically switching water uptake strategies, tapping either shallower or deeper soil layers, depending upon availability (Wang et al., 2019). In the process of transporting water from roots to leaves, the lower water conductivity of the canopy, branches, and leaves influences plant water status (Turner, 1982; Nissanka et al., 1997). When the amounts water absorbed by the root system are insufficient to maintain plant growth, plants may experience severe water shortages and succumb to physiological damage (Huang and Zhang, 2016). To mitigate such water deficit-induced damage, some plants can absorb condensate water through their leaves, thereby enhancing plant water potential (Hill et al., 2015; Dawson and Goldsmith, 2018; Berry et al., 2019; Hill et al., 2021).

Ephemeral plants are particularly important members of the arid desert flora (Goldblati, 1978; Ludwig et al., 1988; Brown, 2003) that have notable community significance (Qiu et al., 2007), playing key roles in ameliorating the effects of wind velocity, stabilizing the sandy landscape, conserving water and soil, and enhancing microhabitats (Wolfe and Nickling, 1993; Wiggs et al., 1995; Qiu et al., 2007). The long-term drought avoidance survival mode of ephemeral plants, based on structure, phenology, physiology, and biochemistry, has enabled these plants to adapt to extremely arid environments, and they typically occur as pioneer species in vegetation successions (Wang et al., 2005). Ephemeral plants, as their name suggests, are genrally short-lived species, with an average lifecycle of approximately 2.5 months. Preferentially exploiting early spring rains, they are characterized by rapid growth and development and contracted phenological stages. Moreover, their root systems tend to be shallow, generally distributed within the 10–30 cm soil layer, and rarely exceeding 40 cm.

Although early studies on ephemeral plants tended to focus primarily on flora and classification (Goldblati, 1978; Ludwig et al., 1988; Brown, 2003), more recently, authors have been turning their attention to the adaptation of ephemeral plants to specific habitats, including studies on the relationships between the physiological and ecological characteristics of these plants and environmental factors (Ehleringer, 1983; Wang et al., 2005; Qian et al., 2007; Qiu et al., 2007). To date, however, comparatively less research has been conducted on the systematic water use strategies of ephemeral plants. To enhance our understanding of the drought resistance mechanisms of ephemeral plants, it is necessary to investigate the water use strategies adopted for the absorption, transport, and utilization of limited water resources. In addition, ephemeral plants may also modify their water use strategies at different stages of growth. For example, in the early stages of growth, the leaves of winter annual plants in the Sonoran Desert have a short-lived high vapor conductivity and water potential, which subsequently gradually decrease in the full bloom stage (Sawada et al., 2002), whereas in contrast, the vapor conductivity and diurnal water potential of the leaves of summer annual plants tend to remain almost constant throughout growth (Ehleringer, 1983). Moreover, different topographical factors may also affect the water use strategies of ephemeral plants, thereby leading to differences in biomass allocation (Zhang et al., 2021).

The Badain Jaran Desert is characterized by a series of mega-dunes with average heights of between 200 and 300 m. During field investigations conducted in the Badain Jaran Desert from June to July 2019, we discovered that many ephemeral plants typically grow in different parts of these mega-dunes. In this habitat, the roots of plants are distributed at some distance above the groundwater table, which lies at a depth of approximately 1.5-2 m in the main concentrated areas of the lake group (Huang, 2018). Precipitation in this region is sparse, with a multi-year average annual precipitation ranging from 50 to 100 mm, mainly falling in the months June to September, and over the past 50 years, the regional climate has shown a warm and dry trend (Hu, 2021); Given that the moisture content in the sand layer in this desert is extremely low (less than 3%) (Cui et al., 2017), it is remarkable that plants are able to grow in this exceptionally arid environment. It is thus of particular interest to determine the mechanisms whereby these plants efficiently utilize the limited water resources during different growth stages and also to established whether ephemeral plants growing at different sites in the dune system adopt different water use strategies. Clarifying the water use strategies of ephemeral plants in different parts of mega-dunes and gaining an understanding of their drought resistance mechanisms would contribute to predicting the future of desert plants under deteriorating moisture conditions, provide a theoretical basis for the screening of vegetation species in sandy areas, and yield scientific evidence that could be used to guide the rational allocation of water resources in sandy areas.

Over recent years, the hydrological cycle of the Badain Jaran Desert region has been a particularly active area of research (Chen et al., 2004; Zhao et al., 2011; Chen et al., 2014; Ma et al., 2016), and in this regard, the distribution of ephemeral plants can provide a good indication of the water content of the sand layer. Accordingly, the study of the plant–water relationships on mega-dunes may provide important insights with respect to the hydrological cycle in this region. In this context, *Ferula bungeana*, a perennial herb in the family Umbelliferae, which grows to heights of approximately 30 cm and has roots of approximately 40 cm in length and up to 8 mm in diameter, is generally distributed in sandy soils in most parts of North China, growing in dunes, sandy land, dry fields, roadsides, and gravel slopes. It is a typical ephemeral plant, with a growing season extending from May to July. It is also distributed among the mega-dunes in the southeastern part of the Badain Jaran Desert, but is rarely found in the flat terrain between mega-dunes. Consequently, we reasoned that by studying the water use strategies of this plant, we might gain important insights on the hydrological processes of mega-dunes. Furthermore, as a representative dune plant, studying the water use strategy of *F. bungeana* would be expected to enhance our understanding of the water use of ephemeral plants in general and contribute to elucidating the drought tolerance mechanisms of these plants.

In this study, we used the stable oxygen isotope technique (iso-source model) to analyze the sources of water used by the ephemeral plant *F. bungeana* on mega-dunes in the Badain Jaran Desert, including the contribution fraction of each soil layer and seasonal variations. Simultaneously, we analyzed the diurnal and seasonal variation characteristics of plant water potential, hydraulic conductivity, and water use efficiency to examine the adaptive mechanisms of *F. bungeana* under natural water stress conditions. Our comprehensive summary of the water use strategies of *F. bungeana* on these mega-dunes will provide a basis for the selection of appropriate species for use in desertification control and effective utilization of plant and water resources. The purposes of this study were to (1) quantify the proportion of soil water absorbed by *F. bungeana* in each soil layer and analyze the seasonal variation characteristics of water absorption; (2) analyze the temporal and spatial variation characteristics of soil water potential and the hydraulic properties of *F. bungeana*, and examine the mechanism of water acquisition by the roots of *F. bungeana*; and (3) determine the temporal and spatial variation characteristics of root, stem, and leaf water potentials and water use efficiency of *F. bungeana*, and summarize the adaptive mechanisms of *F. bungeana* under conditions of long-term water stress.

## Materials and methods

### Study sites

**Figure 1:**
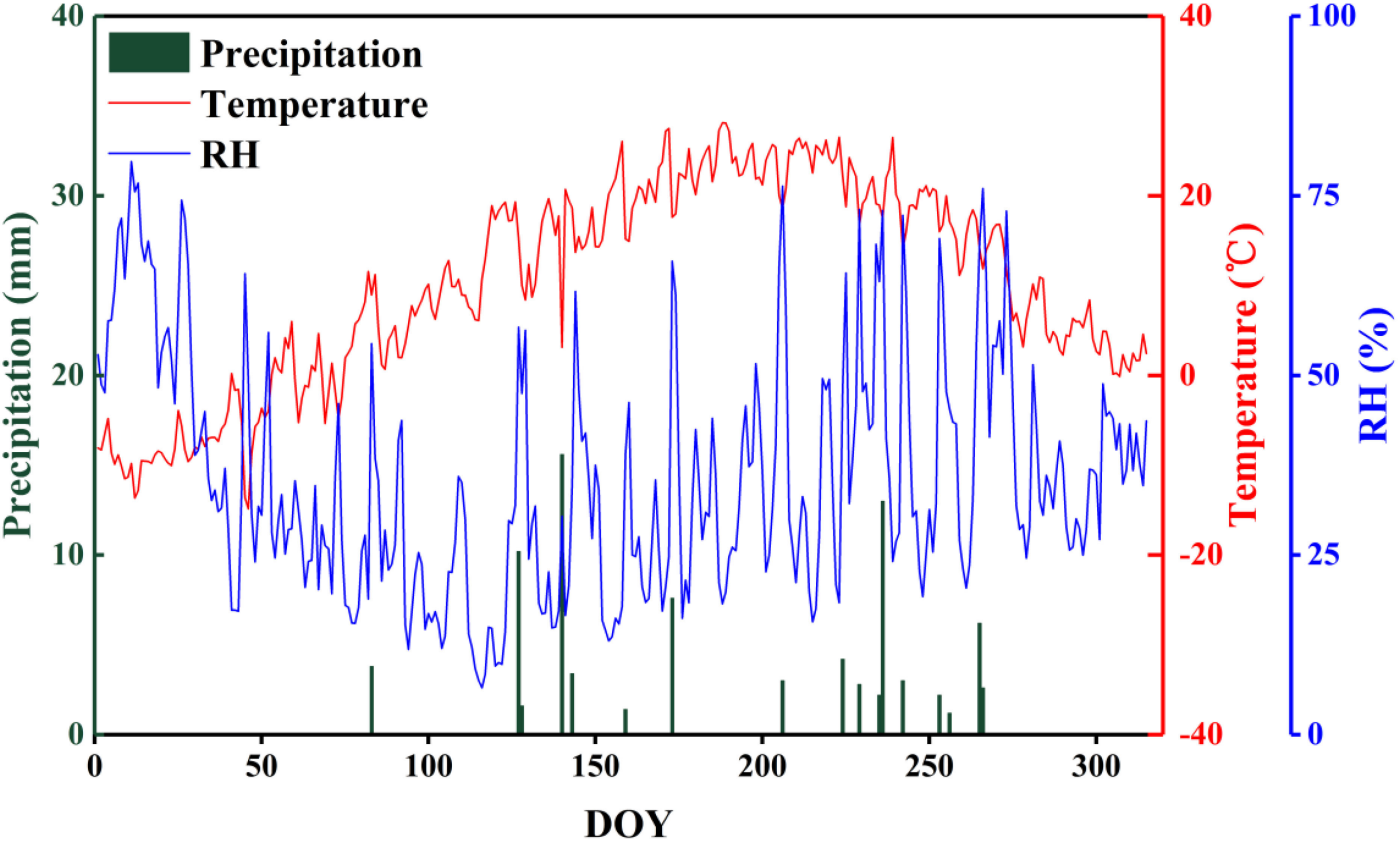
The distribution of monthly precipitation, temperature, and relative humidity during 2020 at the experimental site in the Badain Jaran Desert.

The study area is located near Lake Badain (39°43′19″N, 102°37′01″E) in the southeast of the Badain Jaran Desert (39°04′15″–42°12′23″N, 99°23′18″–104°34′02″E), which lies in the western part of the Alxa Plateau, Inner Mongolia, China. The landscape in this region is characterized by the coexistence of mega-dunes and lakes, the former of which account for more than half of the total desert area, and typically range in height from approximately 200 to 300 m, although can reach a maximum of close to 500 m. The lakes cover an area of 23 km^2^, with a total of 144 lakes, 72 of which still contain water. The coverage and species diversity of the vegetation in this area are generally low, and mainly comprises xerophytic and ultra-xerophytic shrubs and semi-shrubs and diverse herb species. The region is characterized by an extremely temperate continental arid climate, with dry and hot summers, cold and windy winters and springs, and an average annual temperature of around 7 to 8°C. The area receives very little precipitation (**Figure 1**), whereas the rate of evaporation is high, leading to water losses substantially greater than the amounts obtained from precipitation. The groundwater level fluctuates between 0.6 and 10.2 m (actual findings in the main concentrated areas of the lake group). The water content of the wet sand layer of mega-dunes is less than 3% (Cui et al., 2017), which limits plant survival, even though the water balance in this area is positive. These characteristics thus tend to indicate that there are certain cyclic transformation relationship among groundwater, lake water, sand layer water, and plant water in the Badain Jaran Desert and various water sources, and for a number of years, the mutual transformation relationships in this region have been a particularly active area of research.

The upper parts of the windward slopes of the mega-dunes are dominated by perennial herbs, whereas the central parts are dominated by shrubs and perennial herbs, and in the lower parts, semi-shrubs and perennial herbs predominate. In addition, annual herbs can be found growing across the entire elevation range. With regards to the leeward slopes, the upper parts are dominated by annual herbs, and perennial herbs predominate on the central and lower slopes. The dominant species of shrub and semi-shrub are *Zygophyllum xanthoxylum* and *Artemisia ordosica*, respectively, whereas *F. bungeana* and *Psammochloa villosa* predominate among the perennial herbs and *Agriophyllum squarrosum* is generally the most abundant species of annual herb.

### Analysis of the δ^18^O of plant water and water sources

**Figure 2:**
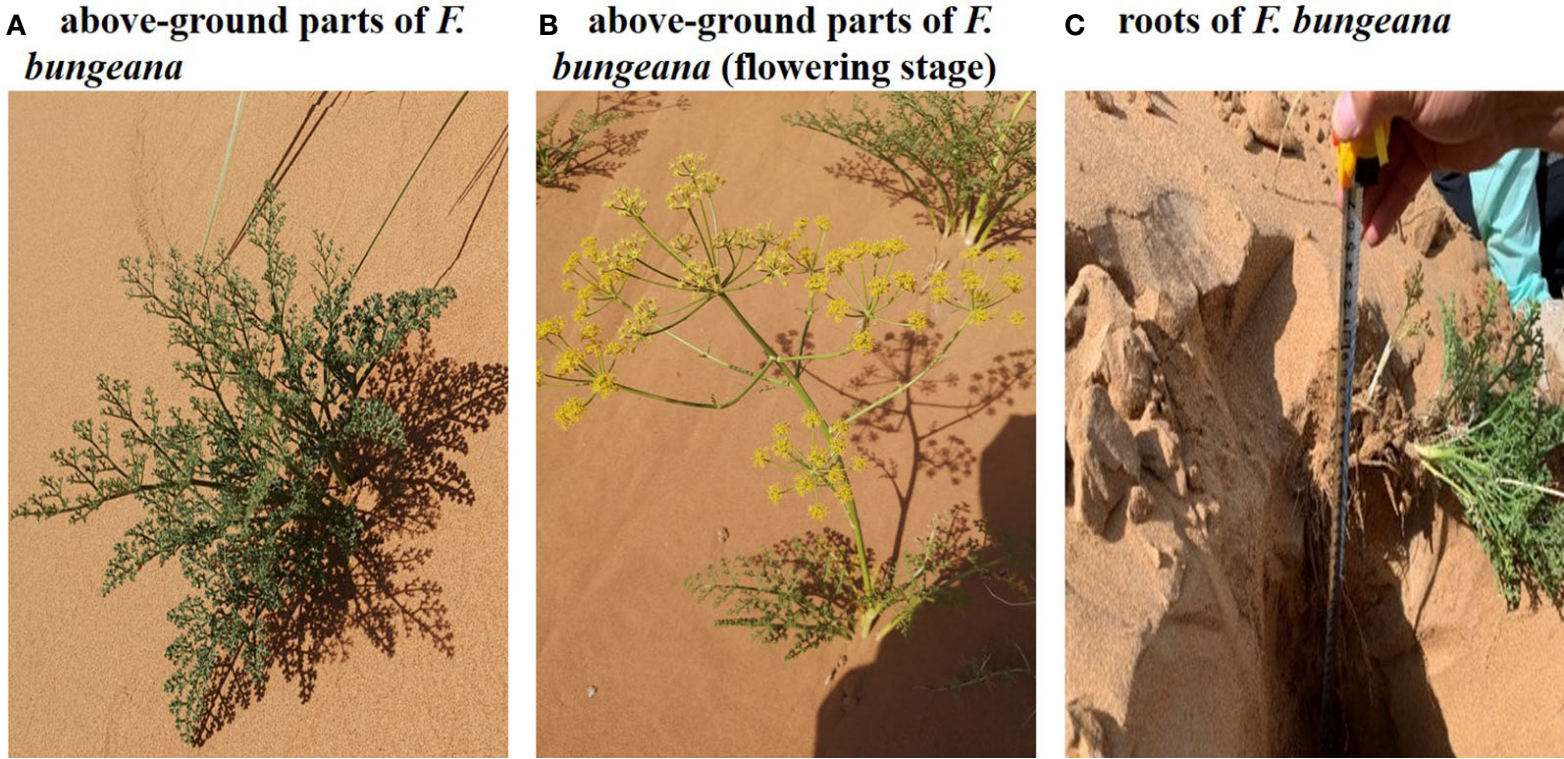
Photographs of *Ferula bungeana* growing on mega-dunes in the Badain Jaran Desert. **(A, B)** present the above-ground parts of Ferula bungeana and **(C)** present the root system of Ferula bungeana.

Experiments were carried out from May to July 2020, during the observation days of which, samples were collected between 06:00 to 09:00 (typically a sunny day) in the middle of each month. The mega-dunes were divided into three sections, the upper, central, and lower sections, and *F. bungeana* growing on these mega-dunes was selected as the focal plant species (**Figure 2**). Within each area, three plants with uniform growth were selected, with three replicate analyses being performed for each sample. The complete root system (**Figure 2C**) was dug out of the sand with a shovel and the rhizosphere soil was removed using the “root shaking method”. Subsequently, the fibrous roots and aerial parts were separated using scissors. The thick roots (the combined part of the rhizomes) were collected, rapidly placed into sampling bottles, sealed with Parafilm, placed in an ice box, brought back to the laboratory, and stored in an ultra-low temperature freezer at -20°C until used for water extraction.

Soil profiles were excavated from sample plots established in different parts of the mega-dunes, and soil samples were collected at different depths, starting from the appearance of the wet sand layer, with a sampling interval of 30 cm and a total sampling depth of 240 cm. Samples for isotopic analysis were collected in plastic vials and sealed with Parafilm, with three replicate samples being collected from each layer. These were rapidly placed in an ice box, and stored at -20°C in the laboratory until used for water extraction. Following sample collection, the excavated sand was backfilled.

For the purposes of isotopic analysis, we initially used a cryogenic vacuum distillation system (Ehleringer et al., 1991; Zhao et al., 2016) to extract water from plant and soil samples at the Key Laboratory of the Inland River Basin, Northwest Institute of Eco-Environment and Resources, Chinese Academy of Sciences. Following extraction, the water samples were filtered through 0.22 μm pore size filters, transferred to 2 mL crimp cap vials, and stored at 4°C until used for isotopic analysis. The δ^2^H and δ^18^O values of the extracted water were determined using an isotope ratio infrared spectroscopy (IRIS) system, comprising a liquid water isotope analyzer (LWIA, 912-0008-1001; Los Gatos Research Inc., Mountain View, CA, USA). The isotopic composition of the water samples was expressed as follows:


(1) $$\delta \text{X}(‰)=(R_{sample}/R_{\text{standard}}-1)\times 1000$$

where *R_sample_* and *R_standard_* are the hydrogen and oxygen isotopic compositions (^2^H/^1^H and ^18^O/^16^O ratios) of the sample and standard water (Vienna Standard Mean Ocean Water, V-SMOW), respectively.

### Quantification of the water sources used by the plants

Taking into consideration the hydrogen isotope fractionation in some xerophytes (Ellsworth and Williams, 2007; Zhao et al., 2016), only δ^18^O values were used for water source analysis and calculations. The iso-source model (Phillips and Gregg, 2003) was applied to estimate the proportional contributions of different water sources to the composition of plant water, with the source increment defined as 1% and the mass balance tolerance defined as 0.1‰. On the basis of the distribution of *F. bungeana* roots and the similarity of δ^18^O values in each soil layer, the potential water sources were divided into the following four parts: soil water at depths of 0–30, 30–60, 60–90, and below 90 cm.

### Soil water potential

Soil water potential was measured using a WP4 dew point potential meter (Decagon, Pullman, WA, USA). Given that the time for which soil has been exposed influences measurements, when excavating soil profiles, fresh soil was collected immediately each time a layer was excavated, and the water potential was measured and recorded.

### Plant water potential

Plant water potential was measured using a pressure chamber water potential meter (Plant Moisture Stress; Corvallis, Oregon, USA). On each measurement day, three measurements were performed at predawn, midday, and during the evening. For measurements, we selected well-developed upper leaves from the middle and upper sections on the sunny side of sample plants, and simultaneously collected branches and uninjured roots from the same plant, and placed these into the pressure chamber to measure their water potentials, with each measurement being repeated three times. During each of the three periods, all measurements were completed within 1 h, and the times of measurement of predawn water potential were adjusted according to the time of sunrise, and was generally completed prior sunrise.

### Plant hydraulic conductivity

Plant hydraulic conductivity was measured based on the water perfusion method using a high-pressure flow meter (HPFM-GEN3; Dynamax Inc., Houston, USA), and was performed simultaneously with our measurements of plant water potential. Measurements of the hydraulic conductivity of the canopy were carried out using the flow meter in “steady mode” at a steady pressure of 350 kPa until attaining a stable water flow. Root hydraulic conductivity was measured using the flow meter in “transient mode”, during which the pressure was rapidly increased from 0 to 500 kPa. On the basis of the pressure and flow, we performed linear regression analysis, with the slope obtained representing hydraulic conductivity. Each of the hydraulic conductivity measurements was performed *in situ* over a period of approximately 10 mins. The entire canopy of sample plants was cut from the main root at a distance of 3 cm above the sandy soil surface and rapidly placed into a water-filled bucket. To prevent air from entering the xylem, the base of the canopy was re-cut underwater, and the hydraulic conductivity of the entire canopy (**Figures 2A, B**) and the entire root system (**Figure 2C**) was measured.

### Measurement of the δ^13^C of plant leaves

Plant leaf samples collected in the field were placed in an oven, deactivated at 105°C for 25 min, and dried at 70°C for 48 h to a constant weight. Having dried, the leaves were ground to pass through a 100-mesh sieve, sealed, and stored in a dry place. The δ^13^C values of plant leaves was determined using an isotope ratio mass spectrometer (IRMS) (DELTA V Advantage; Thermo Fisher Scientific, Bremen, Germany), with the isotopic composition expressed as in formula (1), with *R_sample_* and *R_standard_* representing the carbon isotopic compositions (^13^C/^12^C ratios) of the sample and standard (Vienna Pee Dee Belemnite, V-PDB), respectively.

### Data analysis

Data analyses were performed using SPSS 21.0 software (SPSS Inc., Chicago, IL, USA), and figures were prepared using Origin 2017 software (OriginLab Corp., Northampton, MA, USA). One-way analysis of variance (ANOVA) was applied to determine differences in the contribution of the fractions of each layer of soil water to *F. bungeana*, soil water potential, and leaf δ^13^C values across months and different sites on the mega-dunes. One-way ANOVA was also used to analyze differences in the soil water potential of different soil layers and to identify the differences in plant water potential, as well as the hydraulic conductivity of different parts of *F. bungeana*. Temporal differences in leaf, stem, and root water potentials, and canopy hydraulic conductivity were analyzed using one-way ANOVA combined with the *post hoc* Tukey’s honestly significant difference (HSD) test. Two-way ANOVA combined with *post hoc* Tukey’s HSD test was further conducted to analyze differences among the predawn, midday, and evening leaf, stem, and root water potentials, and predawn, midday, and evening canopy and root hydraulic conductivities of *F. bungeana*, among months and at different sites on the mega-dunes. The significance level for statistical analyses was set at 0.05. Pearson’s correlation analysis was conducted to determine potential associations among the water uptake fraction, soil water potential, and root hydraulic conductivity (plant water potential, soil water potential, and hydraulic conductivity or water uptake fraction and water use efficiency).

## Results

### Temporal and spatial variation in water sources

**Figure 3:**
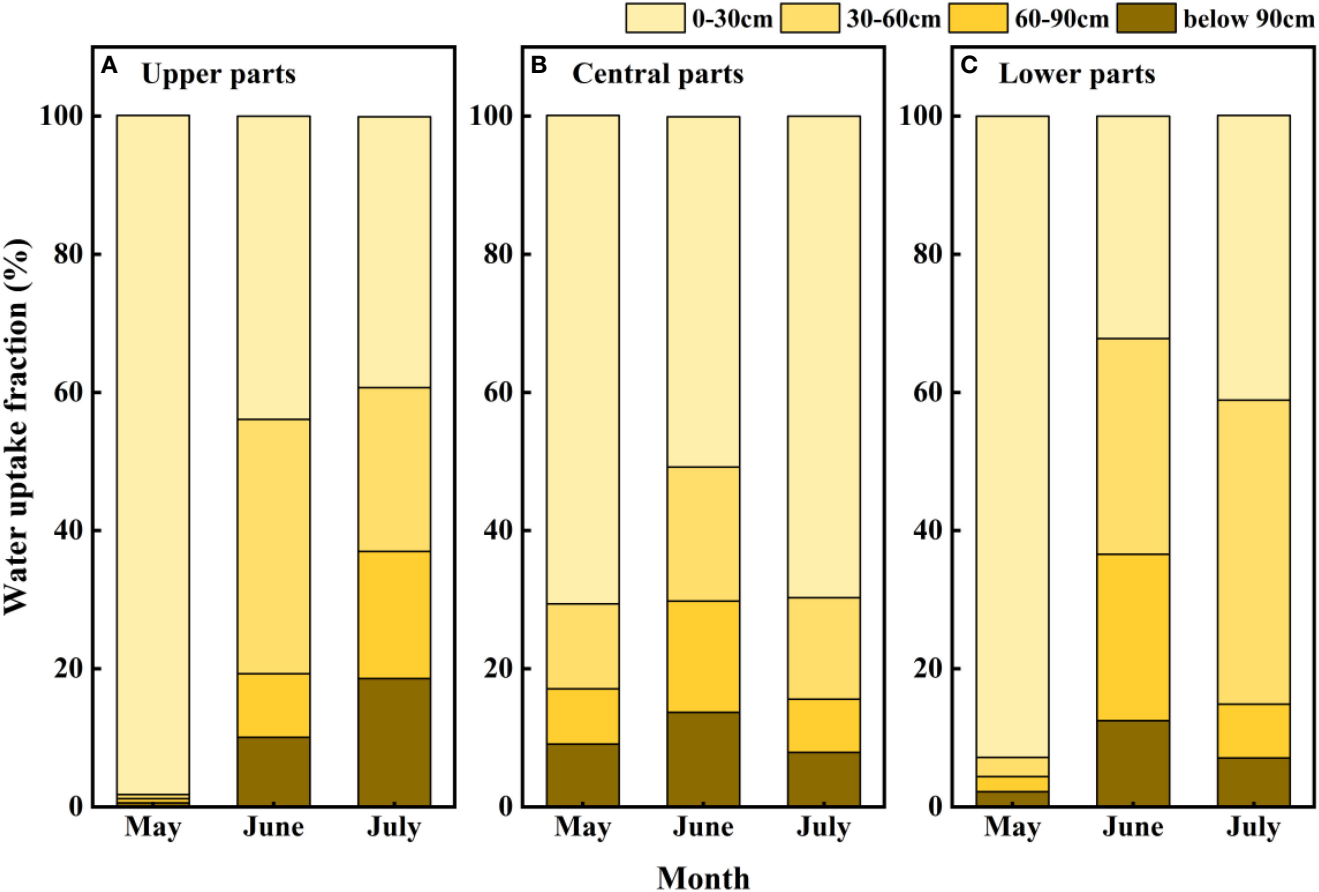
Seasonal variations in water uptake proportions from different soil layers for *Ferula bungeana* growing at different sites on mega-dunes. **(A–C)** present data for the upper, central parts, and lower parts of mega-dunes, respectively. The data were obtained using the iso-source model.

The iso-source model revealed that *F. bungeana* can simultaneously extract water from four zones in the soil profile, although the relative amounts of water extracted from these four sources showed a monthly variation. As shown in **Figure 3**, a majority of the water absorbed by *F. bungeana* (80.47% ± 4.28%) was obtained from the 0–60 cm soil layer. Measurements obtained in May revealed that the major water source used by *F. bungeana* in this month was that in the 0–30 cm layer (87.27% ± 8.43%), although there was a significant reduction in the fraction absorbed from this layer in June (42.27% ± 5.40%) and July (50.03% ± 9.85%) (*P<* 0.05). In contrast, there was a significant increase in the proportional contribution of soil water from the 30–60 cm soil layer (*P<* 0.05) from May (5.23% ± 3.59%) to June (29.13% ± 5.13%), followed by a non-significant reduction in July (27.47% ± 8.67%) (*P* > 0.05). Similar temporal variation was detected with respect to the proportional contributions of soil water from the 60–90 cm soil layer (May, 3.6% ± 2.25%; June, 16.47% ± 4.31%; July, 11.30% ± 3.55%). However, we detected no distinct temporal differences in the percentage contribution of soil layers below 90 cm (*P* > 0.05). We also established that the proportion of soil water contributed by the 0–30 cm layer differed significantly from that derived from the other assessed layers (*P<* 0.001), whereas we detected no pronounced differences in the proportional contributions of different soil layers to *F. bungeana* growing at different sites on the mega-dunes (*P* > 0.05) (**Figure 3**).

**Figure 4:**
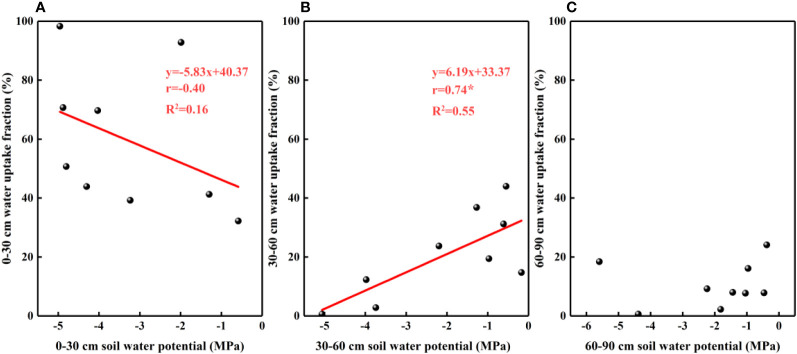
The relationship between soil water uptake fraction and soil water potential. **(A–C)** present data for the 0–30, 30–60, and 60–90 cm soil layers, respectively.

**Figure 5:**
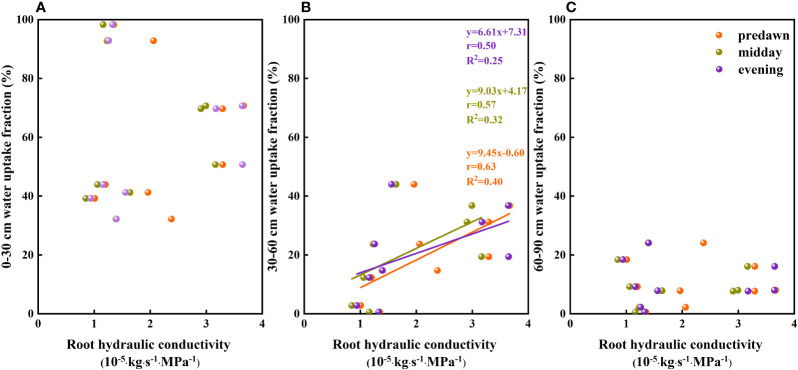
The relationship between the soil water uptake fraction and root hydraulic conductivity of Ferula bungeana. **(A–C)** present data for the 0–30 cm, 30–60 cm, and 60–90 cm soil layers, respectively.

The results of our analysis of the relationship between soil water potential and water source ratio indicated that the 0–30 cm fraction of soil water absorbed by *F. bungeana* was negatively correlated with the water potential of this layer (**Figure 4A**), whereas the absorption fraction of the 30–60 cm soil water was significantly positively correlated with the corresponding water potential (*P<* 0.05)(**Figure 4B**). However, we detected no pronounced association, either negative or positive, between the absorption percentage of the 60–90 cm soil water and the corresponding water potential (**Figure 4C**). Analysis of the relationships between the root hydraulic conductivity and water source ratio of *F. bungeana* revealed that there were no significant correlations between the absorption fractions of the 0–30 cm and 60–90 cm soil water and root hydraulic conductivity (**Figures 5A, C**), whereas the percentage absorption from the 30–60 cm soil layer was positively correlated with root hydraulic conductivity, regardless of the time of day when measurements were obtained (predawn, midday, or evening) (**Figure 5B**).

### Temporal and spatial variation in plant and soil water potentials

**Figure 6:**
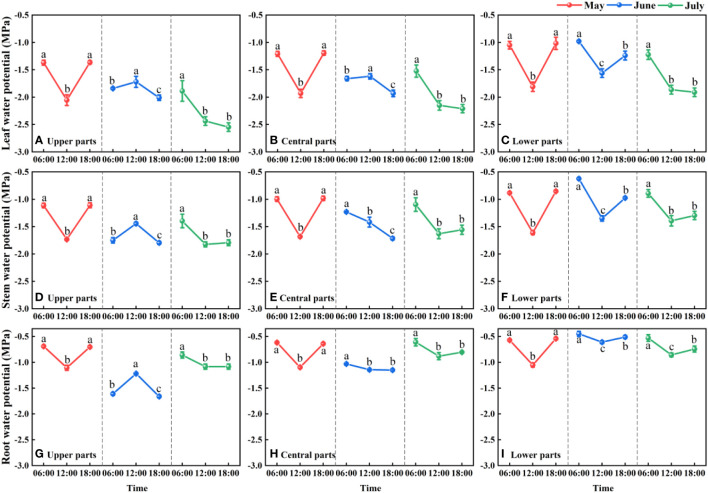
Daily and seasonal variations in the leaf **(A-C)**, stem **(D-F)**, and root **(G-I)** water potentials for Ferula bungeana growing on mega-dunes in the Badain Jaran Desert during the 2020 growth season. Data are presented as the means ± 1SE. Different lowercase letters indicate significant differences in water potential at different times within the same sampling day at the P < 0.05 level of significance.

We detected distinct differences in the water potential of the different parts (root, stem, and leaf) of *F. bungeana* (*P<* 0.05), and established that water was transported upward along a water potential gradient from root to stem to leaf. Root, stem, and leaf water potentials showed the same diurnal and monthly fluctuations. For *F. bungeana* plants measured on the same sampling day at the same mega-dune sites, we detected significant differences in plant water potentials with time (*P<* 0.05), with the diurnal variations in plant water potentials showing a clear monthly variation. Significant monthly variations in the plant water potential at the same time were detected at all mega-dune sites (*P<* 0.05). Additionally, we found that the whole-plant water potentials of *F. bungeana* did not differ among the mega-dune sites (*P* > 0.05) (**Figure 6**).

**Figure 7:**
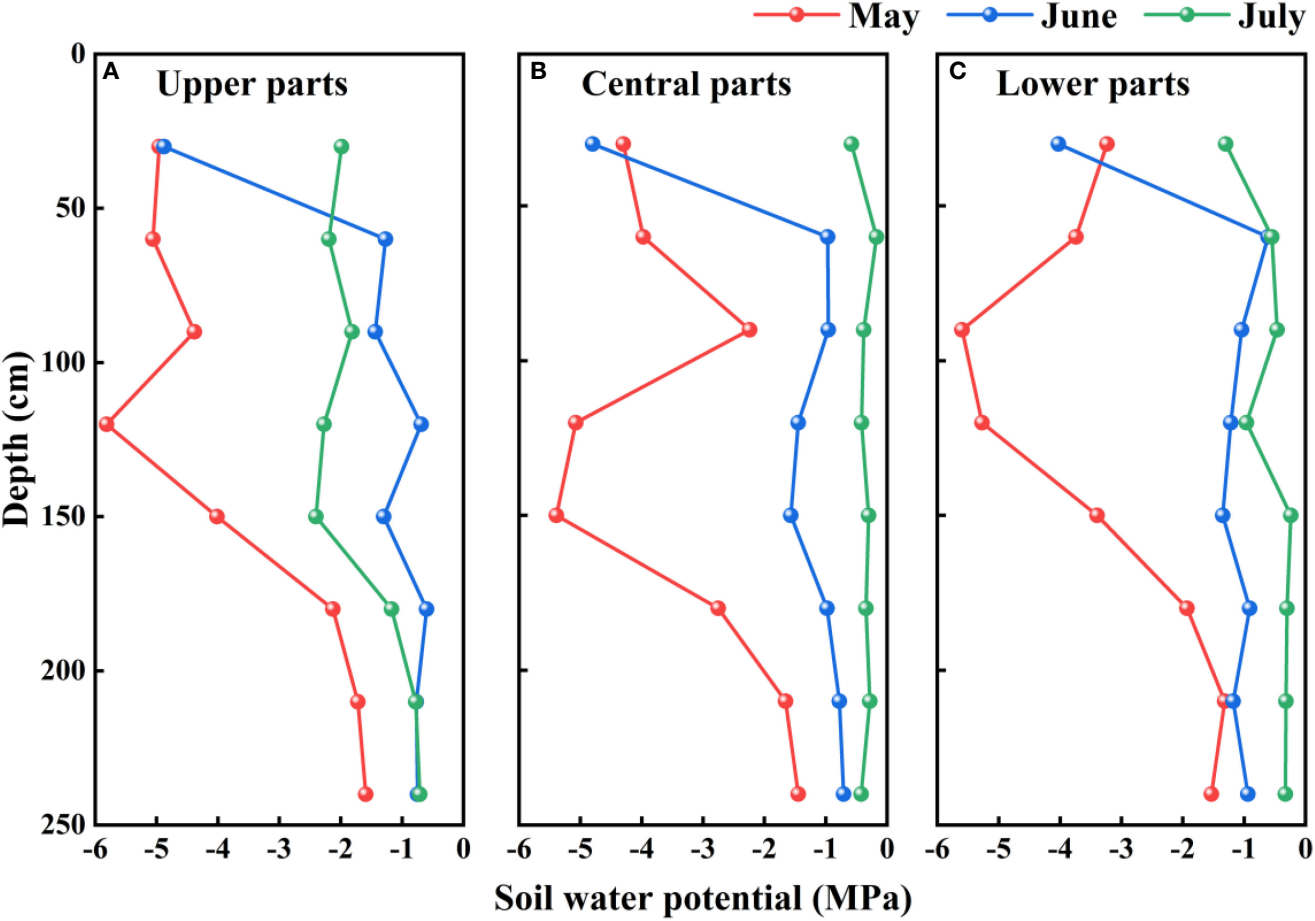
Vertical distribution of the soil water potentials on mega-dunes in the Badain Jaran Desert during the 2020 growth season. **(A–C)** present data for the upper, central, and lower parts of mega-dunes, respectively.

At all assessed sites on mega-dunes in the Badain Jaran Desert, we detected significant temporal and spatial variation in soil water potential in different months (*P<* 0.05) and at different soil depths (*P<* 0.05). The soil water potential in the upper soil layers showed strong monthly differences, with lower values in May and higher values in June and July. Comparatively, the soil water potential in the deeper soil profile fluctuated minimally across months and the water potential values were higher than those close to the soil surface. However, no pronounced spatial differences were detected in soil water potential across the sites on mega-dunes (*P* > 0.05) (**Figure 7**).

### Temporal and spatial variation in plant hydraulic conductivity

**Figure 8:**
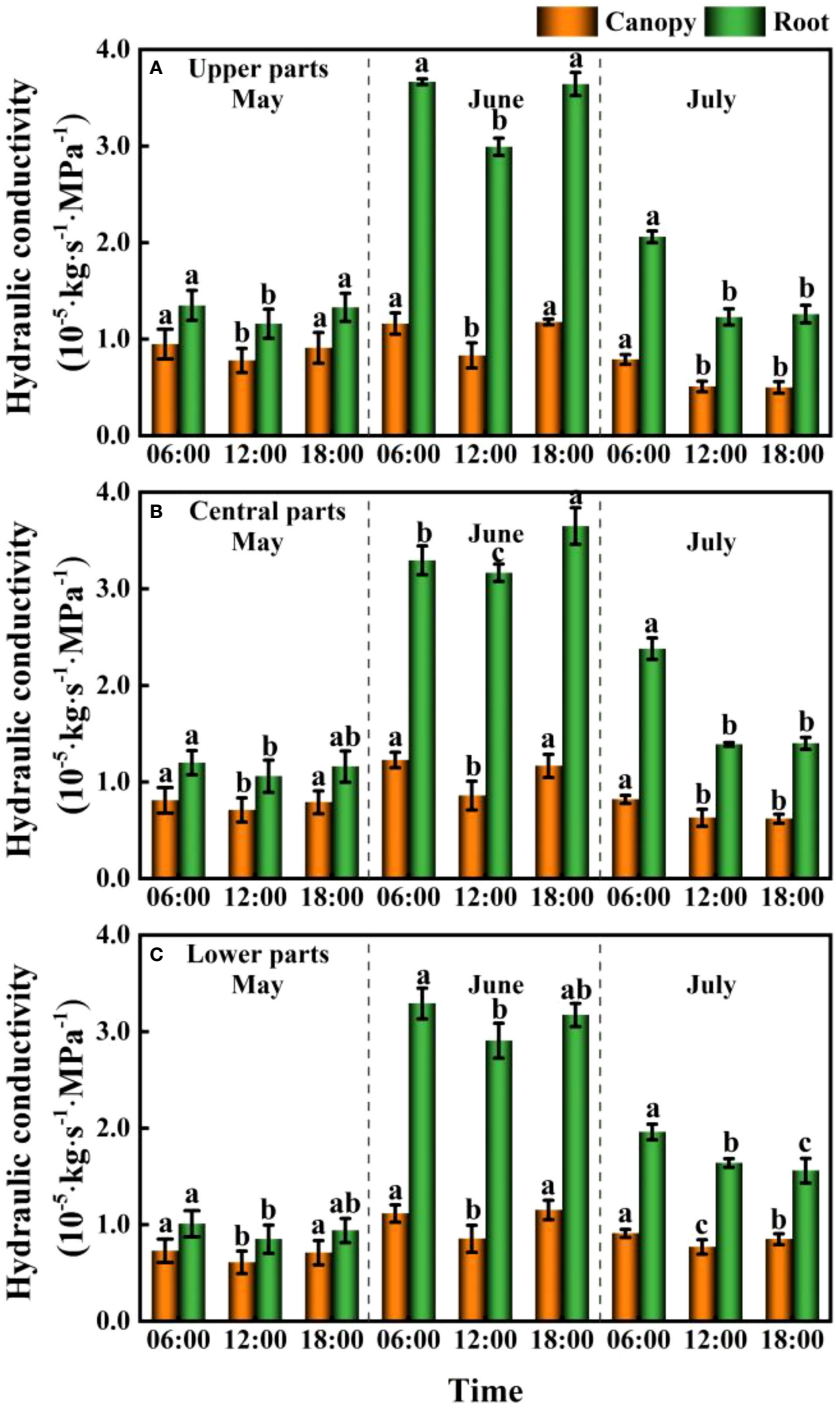
Daily and seasonal variations in the canopy and root hydraulic conductivity for Ferula bungeana growing on mega-dunes in the Badain Jaran Desert during the 2020 growth season. **(A–C)** present data for the upper, central, and lower parts of mega-dunes, respectively. Data are presented as the means ± 1SE. Different lowercase letters indicate significant differences in hydraulic conductivity at different times within a single sampling day at the P < 0.05 level of significance.

We detected significant differences between the canopy and root hydraulic conductivities of *F. bungeana* (*P<* 0.001), with values obtained for the hydraulic conductivity of roots generally being considerably greater than those obtained for the canopy. In addition, diurnal variations in hydraulic conductivity were significant in roots and canopies (*P*<0.05). Pronounced monthly differences were observed in plant hydraulic conductivity (*P<* 0.001), with the largest overall values being recorded in June. Contrastingly, there appeared to be no significant differences in the hydraulic conductivity of *F. bungeana* growing at the different mega-dune sites (*P* > 0.05) (**Figure 8**).

### Temporal and spatial variation in the plant water use efficiency

**Figure 9:**
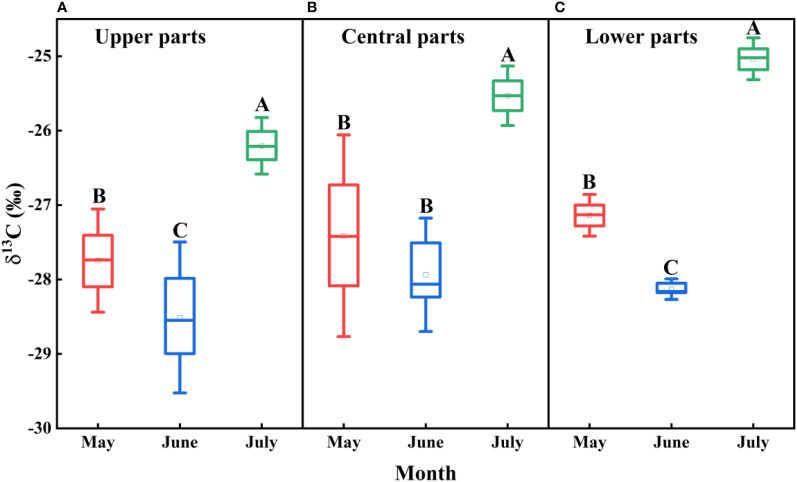
Spatio-temporal variations in d13C values for Ferula bungeana growing on mega-dunes in the Badain Jaran Desert during the 2020 growth season. **(A–C)** present data for the upper, central, and lower parts of mega-dunes, respectively. Data are presented as the means ± 2SD. Different uppercase letters indicate significant differences in d13C values among months at the P< 0.05 level of significance.

There were significant monthly differences in the leaf δ^13^C values of *F. bungeana* growing at all mega-dune sampling sites (*P<* 0.001), with the largest value recorded in July (-25.59‰ ± 0.27‰), the smallest value in June (-28.19‰ ± 0.18‰), and an average value of -27.07‰ ± 0.20‰. However, whereas the differences detected in July were significant (*P<* 0.001), those measured in May and June were not significant (*P* > 0.05), and overall there were no distinct differences in the leaf δ^13^C values of *F. bungeana* among the mega-dune sites (*P* > 0.05) (**Figure 9**).

## Discussion

### Variation in water sources

The fact that *F. bungeana* growing on the slopes of mega-dunes in the Badain Jaran Desert draws water primarily from the 0–60 cm soil layer (**Figure 3**) is consistent with the distribution of its root system, which extends to maximum depths of approximately 50 cm below the dry sand layer. This pattern of water usage is also comparable to that observed for the annual herb *Agriophyllum squarrosum* (Li et al., 2019). Interestingly, however, we also found that *F. bungeana* can utilize a certain proportion of soil water at depths below 90 cm. Given that its roots do not penetrate to these depths, we speculate that the water usage of this plant may be facilitated by the hydraulic lift mediated by *Zygophyllum xanthoxylum* growing in the same habitat (Wu, 2010). In this scenario, deep soil water absorbed by the roots of *Z. xanthoxylum* is released into the upper soil layers, wherein it can be utilized by the shallow branching roots of *F. bungeana*. In addition, *Artemisia ordosica*, which grows in the same dune habitat, utilizes soil water at depths down to 120 cm (Qin et al., 2022), thereby to a certain extent, drawing upon the water sources of *F. bungeana*. Consequently, there may exist a certain degree of competition between *F. bungeana* and *A. ordosica* for limited resources. During the dry season, however, these three species tend to be dependent on soil water sourced from different soil layers, which could be regarded as an effective strategy for hydrological niche segregation, the phenomenon whereby plants are able to coexist in the same habitat by utilizing different sources of water. This partitioning of resources thus serves an important mechanism facilitating plant coexistence in arid water-limited ecosystems (Moreno-Gutiérrez et al., 2012; Wu et al., 2014; Silvertown et al., 2015; Tiemuerbieke et al., 2018; Wu et al., 2019).

The iso-source model adopted in the present study indicated that the water sources exploited by *F. bungeana* show clear temporal variations. In May, *F. bungeana* mainly extracts water from the 0–30 cm soil layer, whereas in June and July, a significant increase was detected in the utilization of soil water at depths between 30 and 60 cm (**Figure 3**). Depending upon water availability, switching water utilization patterns between different soil layers is a necessary strategy enabling plants to tolerate drought conditions in water-deficient ecosystems (Hasselquist et al., 2010; Wang et al., 2019). Seasonal variations in plant water sources are associated with differences in multiple factors, notably soil water content, precipitation, transpiration, and competitive interactions at different growth stages (Zhao et al., 2019). In arid habitats, the upper soil water is predominantly replenished by precipitation, and plants can absorb water from these layers *via* aquaporins (Tan et al., 2018; Wu et al., 2019). With the progression of growing season, as the amounts of precipitation decline and temperatures begin to rise, there is a gradual depletion of soil moisture content, thereby resulting in the dehydration or death of fine roots distributed in the upper soil layer (Barbeta et al., 2015). Therefore, although soil water availability and root activity to a large extent determine the ability of plant roots to obtain water (Wu et al., 2014; Warren et al., 2015; Phillips et al., 2016; O'Keefe et al., 2019), the rooting depth determines the depth of soil from which plants can extract water (Zencich et al., 2002). In the present study, we established that the ratio of soil water absorbed by the roots of *F. bungeana* was largely determined by the water potential of the 30–60 cm soil layer.

However, we established that for *F. bungeana*, the percentage utilization of soil water was highest in the 0–30 cm soil layer, and was not restricted by either root hydraulic conductivity or the soil water potential of this layer, which consequently posed the question as to which absorption path is operational under these conditions. The findings of numerous studies have revealed that dew uptake by plants occurs in most deserts where water is limited by the scarcity of precipitation (Hill et al., 2015; Dawson and Goldsmith, 2018; Berry et al., 2019; Hill et al., 2021). For example, Hill et al. (2015) found that some dominant desert plants derive approximately 50% of their water requirements from dew (Hill et al., 2015), and several studies have provided evidence to indicate direct entry of dew water into leaves (Cavallaro et al., 2020), water uptake pathways into leaves through the cuticle (Gouvra and Grammatikopoulos, 2003; Goldsmith et al., 2017), and water uptake through stems (Breshears et al., 2008; Mason et al., 2016). Hill et al. (2021) also demonstrated that desert plants utilize dew on leaves (including direct absorption of dew by leaves and root absorption of dew falling onto the surface soil), transfer this water to the roots, and subsequent transport the water to the stems *via* different routes (Hill et al., 2021). It is thus conceivable that the input of dew with the same isotopic values as the upper soil causes a negative correlation between the proportion of water absorbed from the 0–30 cm soil layer and the soil potential of this layer, whereas the direct absorption of water *via* the leaves would account for the lack of correlation between the water source and hydraulic conductivity of the root system. However, more in-depth analysis will be necessary to clarify the underlying mechanisms.

### Variations in plant and soil water potentials

Soil water potential fluctuates according to depth (**Figure 7**). The upper soil layers are strongly affected by precipitation and temperature, and the soil moisture content of these layers can undergo considerable fluctuation (Wu et al., 2019). Between May and July, precipitation in this region is typically low, with quantities insufficient to infiltrate downwards (Chen et al., 2014), whereas temperatures are high and there is a pronounced evaporative loss, thereby contributing to a substantial depletion of upper soil moisture. In recent years, studies have shown that air humidity, that is, the absorption of atmospheric water vapor, also has a notable influence on the upper soil moisture in dryland ecosystems (Kosmas et al., 1998; Kosmas et al., 2001; McHugh et al., 2015). Comparatively, deeper soil is influenced to a far lesser extent by environmental factors, and consequently, the levels of soil moisture in these deeper layers tend to be higher and less prone to fluctuate than those in shallower soils (Wang et al., 2017). From the perspective of the water potential gradient, we determined that water in the wet sand layers of mega-dunes is derived from groundwater, which migrates upward by evaporation as a thin film of water (Chen et al., 2004; Chen et al., 2014), thereby leading to a greater isotopic enrichment of the surface soil water (Qin et al., 2022). This process tends to run counter to the idea that local precipitation can infiltrate through mega-dunes to recharge lakes or groundwater (Zhao et al., 2011; Ma et al., 2016). Some experts also believe that the water in mega-dunes originates from the condensation of water vapor from the lakes (Zhao et al., 2011). Given these conflicting views, the hydrological cycle of the Badain Jaran Desert continues to be a subject of active debate.

Plant water potential can indirectly reflect the water status of plants, and its levels indicate the extent to which plants absorb water from the soil or adjacent cells to ensure normal physiological activities (Schütz et al., 2002). In the present study, monthly differences in the diurnal patterns of plant water potentials (**Figure 6**), might be associated with the regional monthly differences in diurnal temperature variation. Plants are dependent on the upward transport of water mediated by the transpiration pull along a water potential gradient (Kim et al., 2014; Zhang et al., 2017), the extent of which is influenced by temperature, thereby affecting the water potential of different parts of the plant. Theoretically, the temperature tends to be highest at midday, when the plant water potential should be lower than that predawn (González et al., 2004). A reduction in plant water potential at midday is beneficial to plants, in that it is conducive to the absorption of water from soil aquifers and reduces water loss to the atmosphere, thereby contributing to plant growth. Conversely, in this study, we found that the plant water potential at midday in June was higher than that recorded predawn (**Figures 6A, B, D, G**). We suspect that this counter-intuitive observation could be attributed to the fact that plants can enhance their water potential by absorbing condensed water (Boucher et al., 1995; Simonin et al., 2009; Eller et al., 2013; Berry et al., 2019; Cavallaro et al., 2020; Yokoyama et al., 2021), and it is conceivable that there might also be a certain hysteresis effect. In addition, foliar water uptake is markedly influenced by environmental conditions, such as wind speed, season, and terrain (Pan, 2001). Monthly differences in these environmental conditions may lead to monthly differences in the water potentials of plants growing at different sites on the mega-dunes surveyed in the present study.

**Figure 10:**
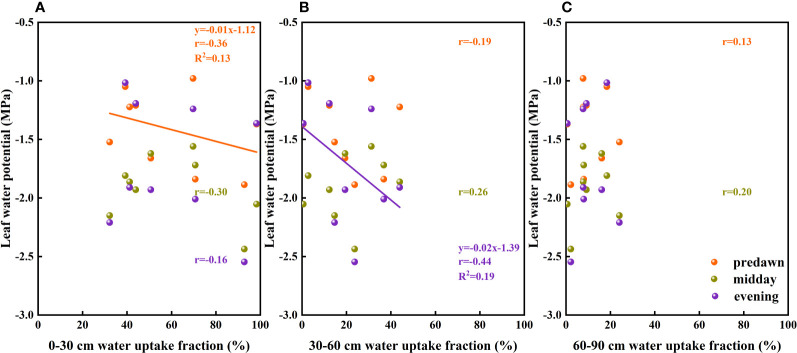
The relationship between leaf water potential and soil water uptake fraction. **(A–C)** present data for the 0–30, 30–60 cm, and 60–90 cm soil layers, respectively.

Leaf water potential is considered to serve as an indicator of the water status of the entire plant (Sibounheuang et al., 2006), which is associated with drought resistance mechanisms (Jongdee et al., 2002; Pantuwan et al., 2002). Differences in water use patterns and water conductance from roots to leaf tips are important for maintaining the leaf water potential of plants (Sibounheuang et al., 2006), and in the present study, we detected different response relationships between leaf water potentials and the absorption fractions of different soil layers (**Figure 10**). These observations would tend to indicate that the water stress experienced by *F. bungeana* is alleviated by the utilization of deep soil water.

**Figure 11:**
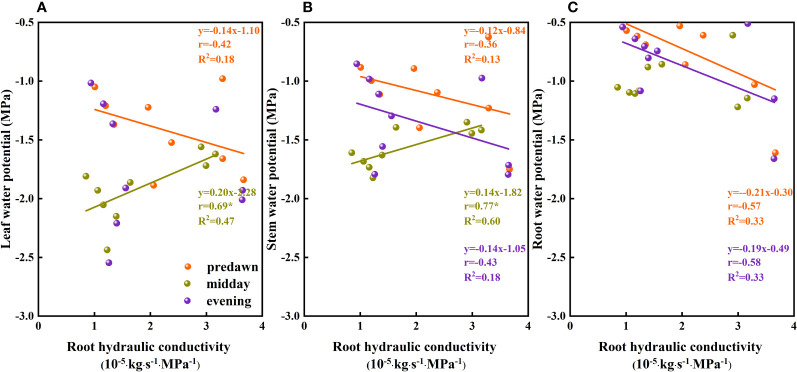
The relationship between the plant water potential and root hydraulic conductivity of *Ferula bungeana*. **(A–C)** present data for the relationships between leaf, stem, and root water potential and root hydraulic conductivity, respectively.

**Figure 12:**
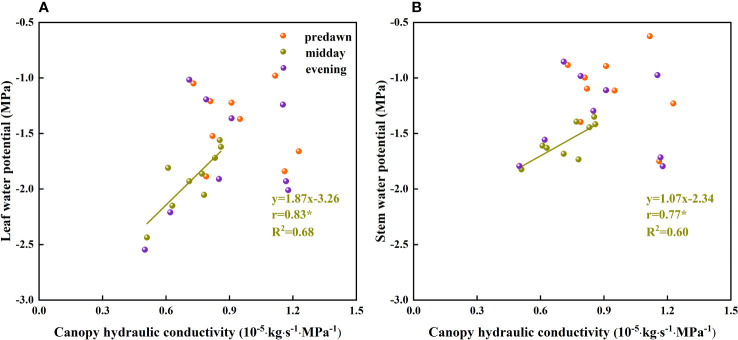
The relationship between the plant water potential and canopy hydraulic conductivity of *Ferula bungeana*. **(A, B)** present data for the relationships between leaf and stem water potentials and canopy hydraulic conductivity, respectively.

When water availability in the upper soil layer is reduced, the movement of water between the soil and roots is restricted by incomplete root–soil contact and an increase in hydraulic resistance, thereby hindering the absorption of water. To ensure uninterrupted water absorption, it is necessary for plants to continuously reduce their water potential. When the plant water potential reaches a certain threshold, plant water sources are gradually separated from shallow soil water and transferred to deep water sources (Wu et al., 2019), which to a certain extent contributes to relieving plant water stress. By extracting water from deep soil sources, plants can restore water potentials. In addition, temporal variation in plant water potential as soil water fluctuates in the same soil layer (**Figure 10B**) could be due to different factors restricting water absorption or the absorption ratio of condensed water or atmospheric water at different times. During the predawn and evening periods of day, leaf and stem water potentials are constrained by other conditions (**Figure 11**). We infer that during the predawn and evening periods of day, leaf and stem water potentials are restricted by canopy hydraulic conductivity, that is, canopy resistance tends to be greater at these times of day (**Figure 12**). If the stem presents a high resistance to water flow, the water uptake capacity of the root system has a limited effect on leaf water potential (Turner, 1982). Even in circumstances in which there is a high stem water potential, there may not be a high leaf water potential if the internal water conductivity of the leaf is low (Nissanka et al., 1997). Similarly, even if there is a high root water potential, if the stem water conductivity is low, there may not be a sufficiently high stem water potential, which in turn influences the leaf water potential. However, in the case of high root hydraulic conductivity, a reduction in root water potential might be attributable to poor soil water conditions and the radial movement of water absorbed by roots, the process whereby water enters the root xylem from the root surface.

### Variations in plant hydraulic conductivity

Within plants, it is necessary for water to overcome a certain resistance to upward conductance. According to the main links of water entering plants from the soil and subsequently diffusing into the atmosphere, the resistance to water conduction can be divided into soil resistance, soil–root contact resistance, root absorption resistance, xylem conductive resistance within roots, internal conductive resistance of the above-ground parts of plants, and water vapor (stomatal) diffusion resistance. Among these, root absorption and stomatal diffusion resistances are the predominant sources of resistance in the below- and above-ground parts of plants, respectively (Shao et al., 1986). The reciprocal of the water flow resistance overcome by plants can be used as an index to characterize and evaluate the water conductivity of the different parts of plants (Yang et al., 2011), namely, plant hydraulic conductivity. The water absorption characteristics of plant roots and the water-conducting capacity of the aerial parts have been shown to have a decisive effect on the survival and growth of plants (Yang et al., 2011), and are also important indicators of the regulation of plant water balance. In the present study, the water transport capacity of the roots was substantially greater than that of the canopy (**Figure 8**), and this stronger water transport capacity of the root system directly influences the rate of water transport in the canopy and the entire plant. The root system is the source of plant water transport, the water absorption and conduction capacity of which play essential roles in determining the water status of the entire plant and the maintenance of plant water balance (Li et al., 2020). In general, the root system and leaves of plants are the parts most prone to embolism, resulting in an overall reduction in plant water conductivity. The most obvious manifestation of such impedance is a V-shaped diurnal variation in plant hydraulic conductivity (**Figure 8**). Monthly variations observed in the hydraulic conductivity of canopies and roots are plausibly associated with changes in growth stage, moisture conditions, and environmental conditions. The internal water transport structural features of plants determine the potential capacity of plant water transport, whereas the external soil moisture status determines the overall level of plant water transport, and environmental factors, such as light, determine instantaneous changes in plant water transport (Si et al., 2007).

As the main driving force for water conduction in plants, water potential is closely associated with plant hydraulic conductivity. Typically, in ecosystems characterized by poor water status, particularly desert ecosystems, the water consumption of plants exceeds the amount of water taken up by roots, thereby resulting in potentially serious deficits. Under conditions of water deficiency, strong negative pressure develop at both ends of the xylem ducts, which exceed the cohesive and adhesive forces between water molecules within the ducts and between water molecules and the duct walls, thereby causing a rupture of the continuous water columns, and entry gas in the xylem ducts. The embolisms thus generated reduce hydraulic conductivity, resulting in a reduction of water potential necessary to maintain a sufficient water balance, which is characterized by the similar diurnal variations in plant water potential and hydraulic conductivity (**Figures 6**, **8**). This can be seen an adaptive water transport strategy adopted by plants to maintain water homeostasis and life activities in specific habitats. However, it remains unclear as to whether a reduction in water potential contributes to a reduction in plant hydraulic conductivity or vice versa.

### Variations in plant water use efficiency

**Figure 13:**
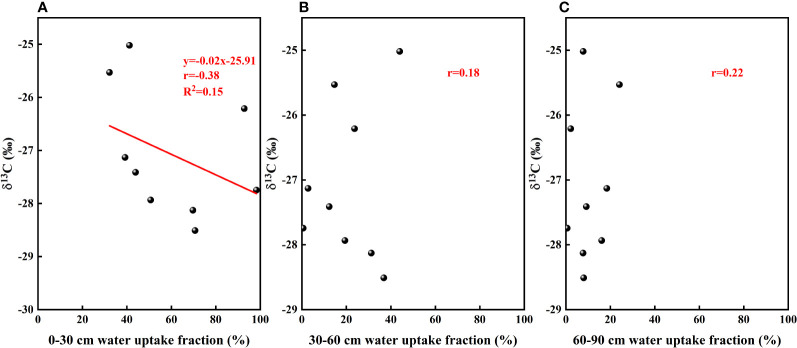
The relationship between leaf δ^13^C values and soil water uptake fraction. **(A–C)** present data for the the relationships between leaf δ^13^C values and the absorption ratio of the 0–30, 30–60, and 60–90cm soil layers, respectively.

Water use efficiency is an important indicator that can be used to characterize the effective water use capacity of plants. In particular, long-term water use efficiency can fully reflect the long-term use of limited water sources and to a certain extent, the capacity of plants to adapt to water stress (Xing et al., 2019). Given that there is a significant positive correlation between the δ^13^C values of plant leaves and water use efficiency, the δ^13^C values are generally used to reflect the water use efficiency of plant leaves. In the present study, we established that the water use efficiency of *F. bungeana* was affected by water use pattern (**Figure 13**). To a large degree, water use efficiency reflects the difficulty plants experience in acquiring water. We found that in response to a reduction in the availability of water in the upper soil layers, plants switch to sourcing water from the deeper layers in the sand dune profile, which is comparatively more difficult. Consequently, plants can resist water stress by enhancing their water use efficiency.

The significant monthly variations in leaf water use efficiency of *F. bungeana* (**Figure 9**) could be associated with one or more of a number of factors, including growth stage, internal factors (e.g., leaf water potential and stomatal activity), and external environmental factors (e.g., light, water, and temperature). Generally, the water use efficiency of plants has been found be higher during the early growth stages than during the latter stages (Yan et al., 1998), which contrasts with our findings in the present study. We suspect that this discrepancy could be attributed to differences in the water use patterns of different plant species at different growth stages. In terms of intrinsic plant factors, plant water potential and stomatal activity both influence water use efficiency by affecting the rates of photosynthesis and transpiration; however, there tends to be a lack of consensus regarding the contribution of these effects. Among the external environmental factors influencing plant development, all factors that influence photosynthesis and transpiration would influence water use efficiency, although to differing extents. Within a certain range of light intensities, light enhancement can effectively improve water use efficiency, and in water-limited environments, plants have been found to be characterized by higher water use efficiency than those growing in water-sufficient environments (Yan et al., 1998). However, beyond a certain water threshold, water stress persists and the degree of water stress aggravation could lead to a reduction in water use efficiency. The water use efficiency of plants also increases in response to increases in atmospheric temperature (Liu et al., 2011). In the present study, we established that variations in the water use efficiency of *F. bungeana* growing at different sites on mega-dunes (**Figure 9**) are mainly associated with the grain size of sand and the depths to which plants are buried in sand in the different parts of the mega-dunes. The lower slopes of mega-dunes are characterized by coarser sand grains and thicker dry sand layers, and the stem sections of *F. bungeana* growing in these areas tend to be buried to a greater depth in the sand than at more elevated dune sites. Notably in this regard, the growth of buried stems supported by sand requires less carbon than that of upright plants of the same height growing above sand level (Pruyn et al., 2000). Consequently, under the same water conditions, the water use efficiency of *F. bungeana* in the lower parts of the mega-dunes was higher than that of plants growing in the more elevated parts of the dunes (**Figure 9C**). Moreover, the unfavorable water relationship in the upper slopes of mega-dunes has been shown to be associated with a lower carbon yield per unit leaf area (Gries et al., 2003), which would account for lower water use efficiency of *F. bungeana* in these upper parts compared with the plants growing at lower elevations (**Figure 9A**).

### Variations in water use strategies

Under conditions of drought stress, plants generally adopt one of two opposing water use strategies: drought avoidance or drought tolerance. Drought avoidance indicates that plants do not encounter drought adversity throughout their growth and development, and thereby evade to detrimental effects of water deficits. Drought tolerance describes the measures adopted by plants to prevent, reduce, or repair the damage caused by water deficit based on metabolic reactions that contribute to maintaining a reasonably normal physiological state when encountering drought stress. A further strategy, drought resistance, refers to the ability of plants to defend themselves against drought and maintain normal physiological and biochemical processes under drought stress conditions. Examples of plants that adopt such strategies include *Lupinus arizonicus*, a drought-avoiding winter annual plant that reduces water loss through stomatal and morphological regulation, whereas the drought-tolerant *Malvastrum rotundifolium* can effectively utilize water, although its stomatal response, leaf movement, and osmotic regulation might contribute to water loss (Forseth and Ehleringer, 1983b). The *F. bungeana* plants examined in the present study were also assessed to be drought tolerant. Among other plants, the early spring ephemerals *Eremopyrum orientale* and *Schismus arabicus* adapt to drought based on higher water absorption and regulation capacities, whereas in *Plantago minuta* and *Senecio subdentatus*, the maintenance of sufficient water levels is achieved by high water-holding capacities and low transpiration intensities (Li and Wang, 1991). In general, ephemeral plants tend to be characterized by higher water use efficiency that can be maintained during the early growth period and when water is abundantly available (Ehleringer et al., 1979; Ehleringer and Forseth, 1980; Forseth and Ehleringer, 1983a), which is consistent with our findings in the present study.

The adaptation of plants to drought stress is a diverse and complex process, and the different water-use strategies adopted by plants at different developmental stages has rarely been reported. In this study, we characterized the water use strategies of *F. bungeana* in terms of water absorption, transport, and use efficiency, although we did not assess the processes associated with water consumption. Accordingly, in future studies, we should ideally focus on water consumption characteristics, including photosynthetic and transpiration rates, to gain a more in-depth understanding of the internal water status and intrinsic adaptation mechanisms of ephemeral plants, and comprehensively analyze their water use strategies.

## Conclusions

The water use strategies of *F. bungeana* growing on the slopes of the mega-dunes in the Badain Jaran Desert are mainly associated with growth stage, and appear to be little influenced by the spatial distribution of plants on different parts of the mega-dunes. During the early stages of growth, *F. bungeana* mainly extracts water from the 0–30 cm soil layer, maintains high water potential and low hydraulic conductivity, and is characterized by high water use efficiency. During the mid-phase of the growth cycle, *F. bungeana* mainly utilizes water obtained from the 0–60 cm soil layer, and maintains moderate water potential and high hydraulic conductivity, although water use efficiency tends to be low. Water is still mainly sourced from the 0–60 cm soil layer during the latter stages of growth, with *F. bungeana* maintaining low water potential and low hydraulic conductivity, although has high water use efficiency. The availability of soil water in the different layers of the mega-dunes is not only determined by precipitation and evaporation, but also by groundwater recharge, condensate recharge, and atmospheric water absorption, and we speculate that condensed water may also be an important source of water for *F. bungeana* in the study area. The proportion of water taken up by *F. bungeana* is limited by soil water availability and root hydraulic conductivity, which affect plant water potential, canopy hydraulic conductivity, and water use efficiency, the latter of which is also determined by carbon allocation.

## Data availability statement

The raw data supporting the conclusions of this article will be made available by the authors, without undue reservation.

## Author contributions

JQ: Conceptualization, Data curation, Investigation, Methodology, Formal analysis, Roles/Writing—original draft, Writing—review and editing. JS: Conceptualization, Data curation, Formal analysis, Funding acquisition, Methodology. BJ: Investigation. CZ: Conceptualization, Data curation, Formal analysis, Investigation. DZ: Investigation. XH: Investigation. CW: Investigation. XZ: Investigation. All authors read and approved the final manuscript. All authors have read and agreed to the published version of the manuscript.
